# Virologic and immunologic failure, drug resistance and mortality during the first 24 months postpartum among HIV-infected women initiated on antiretroviral therapy for life in the Mitra plus Study, Dar es Salaam, Tanzania

**DOI:** 10.1186/s12879-015-0914-z

**Published:** 2015-04-08

**Authors:** Matilda Ngarina, Charles Kilewo, Katarina Karlsson, Said Aboud, Annika Karlsson, Gaetano Marrone, Germana Leyna, Anna Mia Ekström, Gunnel Biberfeld

**Affiliations:** Department of Obstetrics and Gynecology, Muhimbili National Hospital, 65000, Dar es Salaam, Tanzania; Departments of Obstetrics and Gynecology, Muhimbili University of Health and Allied Sciences, 65001, Dar es Salaam, Tanzania; Department of Microbiology and Immunology, Muhimbili University of Health and Allied Sciences, 65001, Dar es Salaam, Tanzania; Department of Microbiology, Tumor and Cell Biology, Karolinska Institutet, Stockholm, Sweden; Public Health Agency of Sweden, Stockholm, Sweden; Department of Laboratory Medicine, Karolinska Institutet, Stockholm, Sweden; Department of Public Health Sciences, Karolinska Institutet, Stockholm, Sweden; Department of Epidemiology and Biostatistics, Muhimbili University of Health and Allied Sciences, Dar es Salaam, Tanzania; Department of Infectious Diseases, Karolinska University Hospital, Huddinge Stockholm, Sweden

**Keywords:** HIV, Virologic failure, Prevention of mother-to-child transmission, Antiretroviral therapy, Drug resistance, Tanzania, Option B+

## Abstract

**Background:**

In the Mitra plus study of prevention of mother-to-child transmission of HIV-1, which included 501 women in Dar es Salaam, Tanzania, triple antiretroviral therapy (ART) was given from late pregnancy throughout breastfeeding up to 6 months postnatally. Here we report findings in a sub-cohort of women with ≤200 CD4cells/μL at enrolment who were continued on ART for life and followed up during 24 months after delivery to determine virologic and immunologic responses, drug resistance and mortality.

**Methods:**

Blood samples for viral load and CD4 counts testing were collected at enrolment and at 3, 6, 12 and 24 months postpartum. HIV drug resistance testing was performed at 12 months. Data analysis included descriptive statistics and multivariate analysis using Generalized Estimated Equations of 73 women with at least two postpartum assessments. The mortality analysis included 84 women who had delivered.

**Results:**

The proportion of women with a viral load ≥400 copies/mL was 97% (71/73) at enrolment, 16% (11/67), 22% (15/69), 61% (36/59) and 86% (48/56) at 3, 6, 12 and 24 months postpartum, respectively. The proportion of women with immunologic failure was 12% (8/69), 25% (15/60) and 41% (24/58) at 6, 12 and 24 months, respectively. At 12 months, drug resistance was demonstrated in 34% (20/59), including 12 with dual-class resistance. Self-report on drug adherence was 95% (64/68), 85% (56/66), 74% (39/53) and 65% (30/46) at 3, 6, 12 and 24 months, respectively. The mortality rate was 5.9% (95% CI 2.5-13.7%) at 24 months. The probability of virologic and immunologic failure was significantly higher among women who reported non-perfect adherence to ART at month 24 postpartum.

**Conclusions:**

Following an initial decline of viral load, virologic failure was common at 12 and 24 months postpartum among women initiated on ART for life during pregnancy because of low CD4 cell counts. A high proportion of viremic mothers also had resistance mutations. However, at 24 months follow-up, the mortality rate was still fairly low. Continuous adherence counseling and affordable means of monitoring of the virologic response are crucial for successful implementation of the WHO Option B+ guidelines to start all HIV-infected pregnant women on ART for life.

## Background

By the end of 2011, women made up 58% of the estimated 23.5 million people living with HIV infection in sub-Saharan Africa (SSA), home to 92% of pregnant women living with HIV worldwide [1]. The proportion of HIV-infected pregnant women in SSA who received antiretroviral (ARV) drugs for prevention of mother-to-child transmission of HIV (PMTCT) was 64% in 2012 [2]. At the end of 2012, 59% of pregnant women in the 21 Global Plan priority countries in SSA who were eligible for antiretroviral therapy (ART) received it for their own health [3]. Programs for PMTCT of HIV are often entry points for ART, hence it is important to understand the sustainability, and virologic and immunologic treatment outcomes among women initiated on life-long ART during pregnancy.

In Tanzania the national HIV prevalence among women 15–49 years of age is 6.2%, but higher in Dar es Salaam, 6.9% [4]. The scale-up of PMTCT has been quite successful in Tanzania leading to a significant reduction in the number of infected infants, from 42,000 in 2001 to 14,000 in 2012. In 2012, 77% of the 97,000 HIV-infected pregnant women in need of PMTCT were enrolled in a PMTCT program. The main challenge is the drop-out from PMTCT in late pregnancy and post-partum [2]. So far PMTCT services are provided in 93% of reproductive and child health clinics (RCH) [5], 98% of pregnant women attend RCH at least once, 43% make at least 4 visits, 85% access HIV testing services but as high as 48% deliver at home [6]. In low-income countries like Tanzania where replacement feeding and caesarean sections are uncommon practices, MTCT rates of HIV can be reduced to 5% or less, when ARV prophylaxis is given during the later part of the pregnancy, delivery and breastfeeding [7-17].

The World Health Organization (WHO) estimates that pregnant women with CD4 counts ≤ 350 cells/μL account for about 38% of all HIV-infected pregnant women and for up to 85% of MTCT of HIV [18]. There is convincing evidence of improved outcomes in adults if life-long ART is started at a CD4 count of ≤350 cells/μL [19,20] instead of at ≤200 cells/μL used earlier [21]. The 2010 WHO PMTCT guidelines for low-income countries recommended ARV prophylaxis including zidovudine (ZDV) or combination ARV prophylaxis from as early as 14 weeks gestation and initiation of continuous ART for maternal health starting at a CD4 count ≤350 cells/μL [18]. The 2013 WHO PMTCT guidelines recommend that all HIV-infected pregnant women irrespective of CD4 cell count should start on lifelong ART (Option B+) [22]. Tanzania has now begun to implement these new guidelines. This will have major implications for the Tanzanian health system including the costs for treatment and monitoring.

The virologic efficacy of ART in SSA is not routinely monitored by plasma viral load assessment except in research environments [23]. Increased scale-up of earlier ART in low-income countries, high drop-out rates, lack of laboratory monitoring for treatment failure and few alternate treatment options raise concerns about undetected drug resistance [24].

This study is nested within the Mitra Plus PMTCT study [8] and aimed at assessing the virologic and immunologic treatment outcomes, development of HIV-1 drug resistance and mortality and to check for determinants of treatment failure during the first two years of follow-up after delivery in a cohort of women on ART for their own health (CD4 count ≤200 cells/μL at antenatal clinic enrollment) in Dar es Salaam, Tanzania.

## Methods

### Study design and settings

The Mitra Plus was an open-label, non-randomized, prospective cohort study as described previously [8]. Enrolment into the Mitra Plus study started in April 2004 and ended in June 2006. The study was conducted at the Dar es Salaam site previously used for the Petra PMTCT trial [25] and the Mitra PMTCT study [7].

### Study population

**Figure 1:**
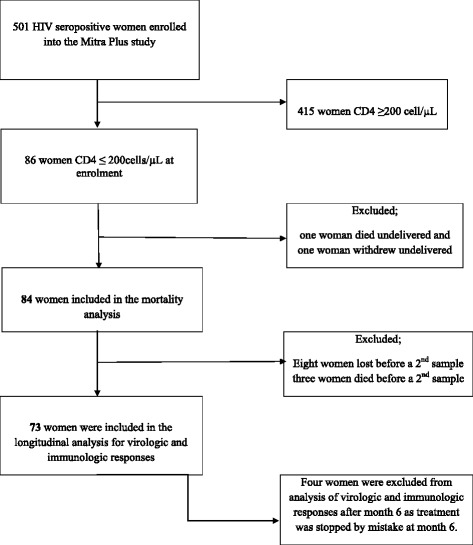
**Flow chart of mothers enrolled with CD4 cell count ≤ 200 cell/μL in the Mitra Plus PMTCT study.**

The Mitra Plus study population consisted of 501 ARV treatment naïve HIV-1 infected pregnant women recruited from four antenatal clinics providing antenatal care services, one each from the three municipals of Dar es Salaam and from the antenatal clinic at the Muhimbili National Hospital in Dar es Salaam. Prior to enrolment, all women signed a written consent form to agree or disagree to participate. For those who could not read and write, fingerprints of the right thumb were used. Out of the 501 women enrolled in the Mitra Plus study, 86 (17%) had a CD4 cell count of ≤200 cells /μL at enrolment. Among these 86 women, two were lost before delivery (one died and the other one withdrew from the study), 8 women who were included in the mortality analysis were excluded from the treatment response analysis due to early loss to follow-up before the month 3 assessment. Three women who died early before they could give a second blood sample post-delivery were also excluded from the treatment response analysis. Hence 84 women were included in the mortality analysis (excluding the two women lost before delivery) and 73 women were included in the longitudinal analysis (Figure 1). An additional four women were excluded from the analysis following their month 6 postpartum visit when ART was stopped by mistake.

### The Mitra Plus study procedure

Enrolled women received normal antenatal care at the Mitra Plus clinic and were asked to attend Muhimbili National Hospital for delivery care. ART was initiated at 34 weeks of gestation or earlier in women with CD4 cells ≤200/μL. Women received ART according to the study protocol: a combination of ZDV 300 mg twice daily + lamivudine (3TC) 150 mg twice daily + nevirapine (NVP) 200 mg lead dose for 14 days which was thereafter increased to 400 mg twice daily. Nelfinavir replaced NVP in women who developed side effects and towards the end of enrolment also for those with a CD4 cell count >200/μL. All women were advised to exclusively breastfeed and to wean abruptly between 5 and 6 months. ART was stopped at 6 months except for the subgroup of women with ≤200 CD4 cells/μL at enrolment who continued ART and this is analyzed in this paper.

Quality of life was determined at enrolment by trained study doctors using the Karnofsky’s score instrument [26]. The scale ranges from 0 – 100, where 100 corresponds to optimal quality of life.

Postnatal follow-up of mother-child pairs was done at weeks 1, 3 and 6, and at months 3, 6, 9, 12, 18, 21 and 24 after delivery. At each visit we performed a clinical examination of the mothers and children and recorded adverse events. Blood samples were drawn for complete blood count, viral load and CD4 cell count assessments at enrolment, month 3, 6, 12, and 24 postpartum. Drug refills and drug adherence counseling was done after every 28 days.

Study nurses conducted home visits to all women who had missed two consecutive postnatal visits to find out and record their reasons for non-attendance and to ask them to come back to the clinic for continuum of care if willing. At the end of the study follow-up women were discharged from the study clinic and referred to care and treatment clinics nearest to their place of residence.

Women came for follow up visits and ARV drug refills every two weeks followed by weekly visits after gestational week 36. After delivery women were given a 30-day drug refill with an appointment after 28 days, providing a two day-margin for drug refill. A self report on drug adherence was given at every clinic visit and categorized as “drugs taken as prescribed yes/no”. Only women who reported not having missed a single dose during the past month were regarded as adherent (“taken drugs as prescribed”).

### Laboratory methods

Screening for HIV antibodies in the pregnant women was done at the recruitment site by trained nurse counselors or health laboratory technologists using the CapillusHIV-1/2 assay (Trinity Biotech, Ireland) for initial testing followed by testing of reactive samples on the Determine HIV-1/2 assay (Abbott Laboratories). Before recruitment into the study, a second sample was collected for confirmatory HIV antibody testing by two consecutive anti-HIV Enzyme-Linked Immunosorbent Assays (ELISAs), Enzygnost anti-HIV 1/2 Plus ELISA (Behring, Marburg, Germany) and Vironostika HIV uniform II plus O ELISA (Biomerieux, the Netherlands) in the Department of Microbiology and Immunology at MUHAS. Sera reactive on both ELISAs were considered HIV-1 antibody positive. Those that gave discordant results between the two ELISA were resolved by a Western blot assay.

Plasma viral load was quantified by the Amplicor HIV-1 Monitor assay version 1.5 (Roche Diagnostics, Indianapolis, IN, USA). The detection limits of the assay using standard protocol testing were 400–750,000 copies/mL. In this study, virologic failure was defined as a detectable HIV-1 RNA load of ≥400 copies/mL at any time during follow up after three months on ART.

Determination of T-lymphocyte subsets was done using the SimulSET flow cytometry method (Becton Dickinson, San Jose, CA, USA) as described previously [27]. Immunologic failure was defined according to the WHO criteria: either a fall of CD4 cell count to pre-therapy baseline or below or a 50% fall of the CD4 cell count from the on treatment peak value, or persistent CD4 levels below 100 cells/μL [28].

Drug resistance testing was carried out on plasma RNA at 12 months after delivery using an in-house method as described previously [29,30]. Resistance to nucleoside reverse transcriptase inhibitors (NRTIs), non-nucleoside reverse transcriptase inhibitors (NNRTIs) and protease inhibitors (PIs) was estimated using the Agence Nationale de Researches Sur le SIDA (ANRS) algorithm (July 2009, version 18).

### Statistical analysis

Data analysis was performed using the STATA software 11 (Stata Corp. College Station, Texas, USA). Mean, median, interquartile range and standard deviation were used for descriptive analysis of numerical variables. Frequencies and percentages were used for categorical variables. Generalized estimating equations were used to estimate the mean viral load and CD4 change across 5 measurement occasions namely: month 0, 3, 6, 12, and 24 and also the association between virologic and immunologic failure with the baseline and clinical characteristics at these time points. The command ‘xtgee’ in STATA was used to run the regression models. The following variables were tested in the regression models; mother’s age (years), mother’s education (years in school), gravidity (number of pregnancies), parity (number of term deliveries), hemoglobin (g/dl), quality of life (Karnofsky’s score 1–100), marital status (married, not married), disclosure of HIV status to partner (no/yes), partners HIV status (negative, not known, positive), and self-reported adherence at 6, 12 and 24 months postpartum (taken as prescribed yes/no). Selection of the mean model and variance-covariance structure was done based on quasi likelihood under the independence model criterion (QIC). Multivariate analysis for drug resistance and mortality were not performed due to small numbers of mothers with this endpoint. For both the mean viral load and CD4 cell change, virologic and immunologic failure models, p-values less than 0.05 were considered significant.

### Ethical approval

The Mitra Plus study protocol was approved by the Institutional Review Boards of Tanzania; National Institute of Medical Research (NIMR) NIMR/HQ/R.Sa/Vol.IX/273, Muhimbili University of Health and Allied Sciences (MUHAS) reference number MU/01/1022 Vol. VXXII/11 which was also renewed in March 2009 with reference number MU/RP/AEC/Vol.XIII/20, and Karolinska Institutet, Stockholm, Sweden (reference number 03–404).

## Results

Table 1 shows the baseline characteristics at enrolment for the 84 women included in the mortality analysis out of whom 73 women were included in the longitudinal analysis. The median age was 29 years (IQR 25–33), 55% were officially married and 7 years was the median length of education. Only 25% had disclosed their HIV status to a partner or a family member and the majority (77%) did not know the HIV sero-status of their partners. The majority of the women were clinically classified as WHO clinical stage 1. The median hemoglobin concentration was 9.9 g/dL, the median CD4 cell count was low, 139 cells/μL, and the median viral load was 66,569 copies/mL (Table 1).

**Table 1:** **Baseline characteristics for Mitra Plus women on ART for life**

**Characteristic**	**All women (n = 84)**	**Analyzed women (n = 73)**
Age in years, median (IQR)*	29 (25–32)	29 (25–33)
**Education years, median**	7	7
**Gravidity, median (IQR)**	2 (1–3)	2 (2–3)
**Parity, median (IQR)**	1 (0–2)	1 (0–2)
**Hemoglobin median (IQR)**	9.9 (8.6-10.9)	9.9 (8.6-10.8)
**CD4 count (cell/μL) at enrolment, median(IQR)**	139 (95–173)	139 (98–174)
**CD4 % at enrolment, median (IQR)**	10 (6–13)	11 (6–13)
**Viral load, median (IQR)**	66,648 (15,253-193,182)	66,569 (15,051-195,289)
^**10**^ **log Viral load mean**	10.77	10.73
**Karnofsky score, median (IQR)**	92 (90–98)	92 (90–100)
**WHO clinical stage at enrolment**		
**Stage 1**	64/84 (76%)	55/73 (76%)
**Stage 2**	10/84 (12%)	9/73 (12%)
**Stage 3**	1/84 (1%)	1/73 (1%)
**Stage 4**	9/84 (11%)	8/73 (11%)
**Marital status**		
**Married**	44/84 (52%)	40/73 (55%)
**Cohabiting**	31/84 (37%)	26/73 (35%)
**Not married**	9/84 (11%)	7/73 (10%)
**Disclosed HIV status to partner/relative**		
**No**	69/83 (71%)	54/72 (75%)
**Yes**	24/83 (29%)	18/72 (25%)
**Partner’s HIV status**		
**Negative**	10/76 (13%)	10/66 (15%)
**Positive**	5/76 (7%)	5/66 (8%)
**Not known**	61/76 (80%)	51/66 (77%)

*(IQR) indicates Inter Quartile Range.

Table 2 shows the proportion of women with detectable viral load (≥400copies/mL) and immunologic failure over a follow-up period of 24 months after delivery. At enrollment, 97% of the pregnant women had a viral load ≥400copies/mL. This proportion decreased to 16% three months postpartum but then increased again to 22% at six months postpartum. Taking month 3 as the reference, the difference in the proportional viral load increase between month 3 and 6 was not statistically significant (p = 0.329). The proportion of women supposed to be on ART for life with detectable viral load significantly increased with time, to 61% at 12 months (p = 0.000) and 86% (p = 0.000), at 24 months. The proportion of women with immunologic failure postpartum also increased significantly to 25% at 12 months (p = 0.032) and 41% (p = 0.035) at 24 months.

**Table 2:** **Proportion of women with viral load ≥400 copies/mL, immunologic failure and self-report on drug adherence in Mitra Plus women on ART for life**

**Time point**	**Number with viral load ≥ 400 copies/ml/number tested (%)**	**Number with immunologic failure/number tested (%)**	**Drug adherence; Number adhering/number tested (%)**
**Enrolment**	71/73 (97%)	-	
**Month 3**	11/67 (16%)	-	64/68 (95%)
**Month 6**	15/69 (22%)	8/69 (12%)	56/66 (85%)
**Month 12**	36/59 (61%)	15/60 (25%)	39/53 (74%)
**Month 24**	48/56 (86%)	24/58 (41%)	30/46 (65%)

At month 12 postpartum, 42% (25/59) had only virologic failure, 7% (4/59) had only immunologic failure, 19% (11/59) had both virologic and immunologic failures while 32% (19/59) had neither virologic nor immunologic failure. At month 24, 46% (26/56) had only virologic failure, 2% (1/56) had only immunologic failure, 39% (22/56) had both virologic and immunologic failure and 13% (7/56) had neither virologic nor immunologic failure.

Table 2 also shows women’s self-report on ART adherence which was 95% (64/68) at 3 months, 85% (56/66) at 6 months, 74% (39/53) at 12 months and 65% (30/46) at 24 months.

At 12 months postpartum, drug resistance mutations could be detected in 34% of 59 women who were available for follow-up at this time point, corresponding to 56% (20/36) of all women with detectable viral load. Among those with detectable drug resistance mutations, 60% (12/20) had resistance to both NRTIs and NNRTIs while 40% (8/20) had resistance to NNRTIs only (Table 3). The most common NRTI-associated mutation was M184V conferring resistance to 3TC, which was demonstrated in 60% (12/20). The most common NNRTI mutations were K103N and Y181C conferring resistance to NVP which were found in 35% (7/20) and 30% (6/20) of the women, respectively.

**Table 3:** **Patterns of reverse transcriptase drug resistance mutations among women with detectable resistance at 12 months postpartum**

**Serial Number**	**Subtype**	**NRTI**	**NNRTI**
**1**	U*	-	K103KN
**2**	C	-	K103KN, H221HY
**3**	A1	-	K103KN**
**4**	A1	M184V	KV106A, Y188CWY
**5**	C	-	V106A
**6**	A1	M184V	Y181C, H221HY
**7**	U	M184V	K101N, Y181C,H221HY
**8**	A1	M184IM	-
**9**	AI		Y181C
**10**	C	M184V	Y181C
**11**	D	-	Y181CY, Y188CY
**12**	U	-	K101Q, E138EGKR
**13**	A1	-	K103N, V108IV
**14**	CRF10_CD	M184V	K103N
**15**	CRF10_CD	M184V	K103N, M230L
**16**	U	M184V	V106AV, G190AG
**17**	C	M184V	K101EK, G190A
**18**	A1	M184V	-
**19**	U	M184IMV	K103KN, Y181CY
**20**	A1	M184V	Y181CY, H221HY

*U **=** unclassified.

** = Minor viral population with resistance associated mutation.

Five women died during the follow up period. One died one week post delivery and the other four women died at month 2,5,14 and 24 post delivery. All women died of HIV-related complications (AIDS). The mortality rate was 5.9% (95% CI 2.5-13.7%) at 24 months.

Table 4 shows the multivariable analysis by Generalized Estimated Equations for the associations between the baseline and clinical characteristics with virologic failure and immunologic failure using month 3 postpartum as reference. The probability of having virologic failure increased tenfold among women who reported non-perfect adherence to ART at month 24 adjusting for time, age, education, marital status, gravidity, parity, HIV status disclosure, partner’s HIV status and hemoglobin concentration (AOR = 10.00; 95% CI: 2.29, 43.66). The probability of immunologic failure was also significantly associated with self-reported non-adherence, but the confidence level was very wide due to small sample size.

**Table 4:** **Generalized Estimated Equation saturated model: Associations between virologic and immunologic failure with baseline and clinical characteristics**

**Variable**	Virologic failure Odds ratio* (OR)	**95% CI**	**p-value**	Immunologic failure Odds ratio* (OR)	**95% CI**	**p-value**
**Month 6**	1.54	0.65-3.67	0.329	3.49	0.48-25.66	0.219
**Month 12**	9.64	2.92-31.86	0.000	6.57	1.17-36.90	0.032
**Month 24**	45.88	10.07-208.96	0.000	9.78	1.16-81.30	0.035
**Age**	0.91	0.78-1.05	0.197	0.78	0.47-1.29	0.333
**Education**	0.92	0.82-1.04	0.191	0.89	0.65-1.00	0.053
**Not married**	2.88	0.46-17.96	0.257	0.02	0.00-18.35	0.271
**Gravidity**	1.49	0.68-3.25	0.317	0.86	0.42-1.87	0.746
**Parity**	0.74	0.30-1.80	0.506	1.08	0.34-3.43	0.898
**Disclosed HIV status**	0.84	0.17-4.18	0.829	4.94	0.58-42.19	0.144
**Partner HIV unknown**	0.75	0.19-2.95	0.675	3.63	0.07-181.81	0.519
**Partner HIV positive**	4.21	0.60-29.38	0.147	1.94	0.03-139.79	0.761
**Hemoglobin**	0.75	0.54-1.03	0.071	0.32	0.07-1.47	0.143
**Not adherent month 6**	2.15	0.57-8.09	0.256	2.85	0.20-41.10	0.442
**Not adherent month 24**	10.01	2.29-43.66	0.002	30.98	1.31-733.91	0.033

*Odds Ratio adjusted for all variables in the table.

## Discussion

We found an unexpectedly high rate of treatment failure and drug resistance in this cohort of HIV-infected women initiated on ART for life during pregnancy in urban Dar es Salaam, Tanzania. The lowest proportion with virologic treatment failure (defined as ≥400 copies/mL), 16% was found 3 months post-delivery, but thereafter increased to 22% at month 6, 61% at month 12 and 86% at 24 months after delivery. Thus, the great majority of women initiated on ART during pregnancy failed to stay on treatment despite needing it for their own health. A high proportion (56%) of mothers with detectable viral load also had resistance mutations at 12 months postpartum. Despite of this, there was a significantly lower two-year maternal mortality rate in this study, 5.9% (95% CI 2.5-13.7%), compared to the 29.9% (95% CI 13.1-46.9%) mortality rate among mothers with ≤200 CD4 cells/μL at enrolment in the previous Petra PMTCT trial at the Dar es Salaam site who only received short-course ART perinatally [31]. The long-term mortality rates in the currently analyzed subgroup with such high failure rates can only be speculated upon.

A review of 89 studies on virologic outcomes among adult African patients on ART showed an overall treatment success defined as a viral load of <400 copies/mL of 78% after 6 months of ART, in 76% after 12 months and in 67% after 24 months [23], but the between study discrepancies were large, varying from 51% to 97% at 12 months and from 46% to 90% at 24 months [23]. The 6 months virologic failure rate in our study (defined as detectable viral load ≥400 copies/mL) was similar to that in the reviewed African studies [23] but higher at 12 and 24 months than in these studies [23]. One possible reason for the high virologic failure rates at 12 and 24 months postpartum in the women in the present study could be that they all participated in a PMTCT study in which women with CD4 counts ≤ 200 cells/μL who were supposed to continue with ART for life, met women with CD4 counts >200 cells/μL who stopped ART at 6 months as per study protocol. The possibility that women shared information in the waiting room and were influenced by peers to discontinue ART cannot be excluded. On the other hand, repeated counseling efforts to promote adherence to life-long ART was carried out at each follow-up visit, more so than in routine ART care. The fact that treatment failure increased after 6 months is likely related to the recommended cessation of breastfeeding at the same point in time. Efforts to adhere to ART among the Mitra Plus women put on ART for life were probably better during the breastfeeding period because women were highly motivated to prevent transmission of HIV to their infants, as supported by the drug adherence self-reports and by qualitative in-depth interviews performed to explore the reasons for treatment failure among women who were to stay on life-long ART [32].

Previous studies of HIV-infected women in resource-limited countries have found high attrition between diagnosis of HIV infection in pregnancy-related services and initiation of ART among those eligible for ART for life [33-35]. A meta-analysis on adherence to ART during and after pregnancy showed that drug adherence was higher during the antenatal period (74%) compared to postnatal period (53%) [36]. Long-term follow up in a community-based ART program in South Africa showed that women who initiated ART during pregnancy had a significantly higher risk of loss to follow-up compared to non-pregnant women [37] while another study from 6 resource-limited SSA countries found similar retention rates and CD4 count responses in HIV-infected women who initiated ART during pregnancy and other adults [38].

We defined virologic failure as a viral load ≥400 copies/mL while previous studies of virologic failure in resource-limited settings often have used a cut-off of 1000 copies/mL [23]. On the other hand, in most high-income settings, virologic failure is defined as a viral load above 20 HIV-RNA copies/mL. Had we used the higher cut-off (≥1000 copies/mL) in our study, the proportion of women with virologic failure at 12 and 24 months postpartum would have remained very high, 53% and 82%, respectively.

As expected virologic failure preceded immunologic failure as a warning indicator of treatment failure and was also associated with self-reported adherence. Several other African studies have also found that CD4 cell count monitoring is a poor indicator for treatment failure [39-43]. A study in a large ART program in the general population in Nigeria showed that immunologic criteria missed almost half of patients with virologic failure [40].

In our study, self-reported adherence correlated but also seriously underestimated the proportion with virologic failure at month 24 postpartum. Several studies have shown that virologic failure in resource-limited countries is associated with incomplete adherence [44-46] and is a strong predictor of resistance [29,44]. Virologic failure has also been shown to be associated with prior NVP-based PMTCT treatment [45,47]. In our study 34% of the women had first line drug resistance at 12 months postpartum, which corresponds to 56% of the women with detectable viral load. Although we did not conduct any baseline analysis to rule out transmitted drug resistance, all women in the Mitra Plus study were treatment naïve. However, a study involving treatment naïve HIV-infected pregnant mothers in Dodoma and youth in Dar es Salaam, showed that the prevalence of ART primary resistant mutations was 11.9% and 9% respectively [48,49]. Assuming similar proportions at baseline among treatment-naïve women enrolled in our Mitra Plus study, it is most likely that the high proportion of drug resistance detectable at 12 months was secondary to suboptimal drug adherence.

The majority of the women with drug resistance in our study had multiple resistance mutations, most commonly M184V, K103N and Y181C. The same resistant mutations were also found in infants of women in the Kisumu PMTCT study [50]. In the Malawi PEPI trial multiclass drug resistance strains were detected in 29.7% of infants of women who were initiated on antiretroviral therapy postpartum [51]. A review of acquired HIV drug resistance in adults in resource-limited settings, including six studies from SSA showed resistance in 11% of patients on ART for 12–23 months [24]. However, a high frequency of drug resistance after one year of first line ART similar to that in our study was reported from Cameroon, 46% [52] and Togo 24.5% [53]. A study of HIV-1 drug resistance after first-line ART failures in six sub-Saharan countries showed that 70% of those with virologic failure (1000 copies/mL) after one year of ART had drug resistant mutations [54]. Accumulation of drug resistance mutations may limit the response to both first and second- line ART regimens and lead to the transmission of drug resistant HIV [55].

Despite close monitoring and follow-up as part of our research study including home visits to all clients who had missed more than two consecutive appointments, 21% (15/73) of the women on ART for life and eligible for follow up after delivery did not return for their 24-month visit. Loss to follow-up and sub-optimal postpartum ART adherence has been major drawbacks to the efforts of combating MTCT of HIV in resource-limited regions [33-36]. This makes it pertinent to discuss how feasible it will be to implement the 2013 WHO guidelines to initiate all HIV-positive pregnant women on ART for life (Option B+). A study done in Malawi, which was the first country to implement the Option B+ recommendations, has shown that 17% of women were already lost to follow-up within six months post-delivery [56].

Thus, there is a clear need for rapid and affordable viral load and drug resistance testing and finding feasible ways to improve drug adherence and minimize loss to follow-up in resource-limited settings.

The main limitation of this study is the small sample size limiting us from observing significant associations so as to draw more firm conclusions. A major strength of the study is the prospective follow-up for 24 months and the availability of viral load and drug resistance information.

## Conclusions

In summary, our findings show that a very high proportion of Tanzanian HIV-infected women with low CD4 cell count who started ART during pregnancy, failed virologically after the breastfeeding period with high risk of drug resistance to standard ART regimens. Counseling for adherence together with use of viral load assessments to check for adherence and treatment response is crucial for achieving successful treatment outcomes. This is especially relevant now when the current WHO guidelines to start HIV-infected pregnant women on ART for life irrespective of CD4 cell count in resource-limited settings (Option B+) are being implemented in several resource-poor countries in SSA including in Tanzania.

## Abbreviations

AIDS: Acquired immunodeficiency syndrome
ANC: Antenatal clinic
ART: Antiretroviral therapy
ARV: Antiretroviral
CD: Cluster of differentiation
DNA: Deoxyribonucleic acid
EFV: Efavirenz
ELISA: Enzyme linked immunosorbent assay
HAART: Highly active antiretroviral therapy
HIV: Human immunodeficiency virus
HIV-1: Human immunodeficiency virus type 1
HIV-2: Human immunodeficiency virus type 2
KI: Karolinska Institutet
MNH: Muhimbili National Hospital
MTCT: Mother-to-child transmission
MUHAS: Muhimbili University of Health and Allied Sciences
NNRTI: Non-nucleotide reverse transcriptase inhibitor
NRTI: Nucleoside reverse transcriptase inhibitor
NVP: Nevirapine
PCR: Polymerase chain reaction
PMTCT: Prevention of mother-to-child transmission
RNA: Ribonucleic acid
SSA: Sub-Saharan Africa
3TC: Lamivudine
UN: United Nations
UNAIDS: Joint United Nations program on HIV/AIDS
UNICEF: United Nations Children’s Fund
WHO: World Health Organization
ZDV: Zidovudine
